# Culture of Primary Ciliary Dyskinesia Epithelial Cells at Air-Liquid Interface Can Alter Ciliary Phenotype but Remains a Robust and Informative Diagnostic Aid

**DOI:** 10.1371/journal.pone.0089675

**Published:** 2014-02-25

**Authors:** Robert A. Hirst, Claire L. Jackson, Janice L. Coles, Gwyneth Williams, Andrew Rutman, Patricia M. Goggin, Elizabeth C. Adam, Anthony Page, Hazel J. Evans, Peter M. Lackie, Christopher O’Callaghan, Jane S. Lucas

**Affiliations:** 1 Primary Ciliary Dyskinesia Centre Department of Infection, Immunity and Inflammation, University of Leicester, Leicester, United Kingdom; 2 Primary Ciliary Dyskinesia Centre, University Hospital Southampton, Southampton, United Kingdom; 3 Clinical and Experimental Sciences, Southampton National Institute for Health Research Respiratory Biomedical Research Unit, University of Southampton, Southampton, United Kingdom; 4 Department of Respiratory Medicine, Institute of Child Health, University College London & Great Ormond Street Children’s Hospital, London, United Kingdom; University of Colorado, Denver, United States of America

## Abstract

**Background:**

The diagnosis of primary ciliary dyskinesia (PCD) requires the analysis of ciliary function and ultrastructure. Diagnosis can be complicated by secondary effects on cilia such as damage during sampling, local inflammation or recent infection. To differentiate primary from secondary abnormalities, re-analysis of cilia following culture and re-differentiation of epithelial cells at an air-liquid interface (ALI) aids the diagnosis of PCD. However changes in ciliary beat pattern of cilia following epithelial cell culture has previously been described, which has brought the robustness of this method into question. This is the first systematic study to evaluate ALI culture as an aid to diagnosis of PCD in the light of these concerns.

**Methods:**

We retrospectively studied changes associated with ALI-culture in 158 subjects referred for diagnostic testing at two PCD centres. Ciliated nasal epithelium (PCD n = 54; non-PCD n = 111) was analysed by high-speed digital video microscopy and transmission electron microscopy before and after culture.

**Results:**

Ciliary function was abnormal before and after culture in all subjects with PCD; 21 PCD subjects had a combination of static and uncoordinated twitching cilia, which became completely static following culture, a further 9 demonstrated a decreased ciliary beat frequency after culture. In subjects without PCD, secondary ciliary dyskinesia was reduced.

**Conclusions:**

The change to ciliary phenotype in PCD samples following cell culture does not affect the diagnosis, and in certain cases can assist the ability to identify PCD cilia.

## Introduction

Primary ciliary dyskinesia (PCD) is a rare inherited multi-genic disorder of cilia, impairing mucociliary clearance. It has a prevalence of approximately 1∶10,000 to 40,000 in Europe [1], [2] with a prevalence as high as 1∶2,200 in certain ethnic backgrounds [3]. Impaired function of motile cilia causes the typical signs and symptoms of PCD. Abnormal mucociliary clearance causes wet cough and rhinitis from soon after birth, recurrent upper and lower respiratory infections, bronchiectasis, sinusitis and otitis media. Approximately 50% of subjects have situs abnormalities and subfertility is common [2]–[4]. To date defects in 20 genes have been shown to cause PCD through the defective coding of proteins that construct the ciliary axoneme or of cytoplasmic proteins that are directly responsible for assembly of ciliary axoneme structures [2], [5].

Subjects with an indicative clinical history should be referred to a specialist PCD service for investigations that may include nasal nitric oxide (nNO) concentration, assessment of ciliary function and ultrastructural axonemal defects [4]; Nasal brush biopsy provides a sample of live and intact ciliated epithelium for assessing ciliary function and ultrastructure. Ciliary function is assessed by high-speed digital video microscopy (HSV) under controlled conditions, which allows ciliary beat frequency (CBF) and detailed beat pattern (CBP) analysis [6]. Ciliary ultrastructure is determined by counting the percentage of structural defects seen by transmission electron microscopy (TEM) of cilia in transverse section [7]. Diagnosis of PCD is often difficult because referred subjects have frequent upper respiratory tract infections with consequent inflammation and damage to the epithelium resulting in secondary ciliary dyskinesia. The sampling itself damages the epithelium, and secondary dyskinesia is frequently seen in completely healthy individuals. The diagnosis is further complicated by some genotypes of PCD being associated with normal ciliary ultrastructure [8]–[12]. Additional methods to help resolve difficult diagnostic cases that have been evaluated include genetic testing [5], which can detect approximately 50% of the PCD cases, radioaerosol mucociliary clearance [13], and immunofluorescence microscopy [14].

Using their ability to regrow healthy ciliated epithelium from an initial sample with a high degree of secondary damage Jorrisen et al [15], [16] first reported the use of cell culture in PCD using a submerged method. The resulting submerged spheroidal cell clusters rotate following ciliogenesis if ciliary motility is normal. Assessment of CBF in suspension culture revealed that some samples from PCD subjects had an abnormal frequency prior to culture which became normal after ciliogenesis, but this had not previously been described in respiratory epithelial cells cultured at an air-liquid interface (ALI) [16]. Culturing followed by ciliation at ALI [17], [18]] has the advantage of yielding more cells than the submerged culture technique, the cilia produced are more numerous and can survive for up to 11 months (unpublished data, Leicester and Southampton), allowing reanalysis of CBF, CBP and TEM ultrastructure of the axoneme, and this has greatly aided the diagnosis of PCD [18].

The PCD diagnostic centres in the UK [19] have found the reanalysis of cilia following ALI culture particularly helpful for confirming that abnormalities seen on the fresh nasal brushing are due to PCD rather than a secondary dyskinesia. The hypothesis of this study was “CBF of the biopsy is the same before and after culture in a large number of PCD cases” This was prompted by our observation that in some patients with PCD we observed a change in ciliary phenotype following culture of epithelial cells. As a consequence, this retrospective study was designed to systematically evaluate the ciliary function and ultrastructure of samples before and after ALI-culture. Here we present a new, definitive and independent observation that ALI culture can, in some cases, cause the ciliary phenotype of PCD to become static.

## Methods

Local and national R&D and ethical approvals were obtained (Southampton and South West Hampshire Research Ethics Committee A CHI395, 07/Q1702/109; University Hospitals of Leicester Research Ethics Committee UHL10049, 06/Q2501/68). All subjects gave written consent for sampling and for their data to be used.

### Subjects and samples

Between 2009 and 2012, 158 subjects referred to centres in Leicester and Southampton had analysis of cilia before and after culture at ALI as part of routine PCD diagnostics. An additional 7 subjects had insufficient cilia for analysis prior to culture, but sufficient following culture at ALI. Leicester and Southampton PCD centres are two of the three national diagnostic centres in England working to share protocols and standards. The HSV and TEM data is audited between the centres to assess accuracy of reporting and to maintain consistency of reporting across the service.

A cytology brush (Olympus Keymed Ltd, 2mm diameter, Southend, UK) was inserted approximately 5 cm into the patients naris and the nasal epithelium was brushed. This method obtained strips of nasal ciliated epithelium, and each brushing contained up to 10,000 cells. Each sample was divided in to three to allow, ciliary functional analysis, fixation for TEM and cell culture. Brushings were only performed if patients had been free from an upper respiratory tract infection for at least four weeks. HSV analysis was conducted on all samples pre- and post- ALI culture. TEM was conducted on all samples with any abnormality of CBP or CBF pre-culture, but not necessarily if function was entirely normal. TEM was generally only conducted following culture if it was expected to aid diagnostic decision making e.g. if the pre-culture TEM sample was insufficient for full analysis, had severe secondary damage or provided an equivocal result.

### High speed video analysis of ciliary function

Protocols were similar across the centres, but equipment differed,(Table 1) hence baseline CBF values were considered to be centre dependent. Cells were suspended in HEPES (20 mM) buffered medium 199 containing penicillin (50 µg/ml), streptomycin (50 µg/ml) and Fungizone (1 µg/ml). Strips of ciliated epithelium (37°C) were imaged using an x100 objective and digitally recorded using a high speed camera. Ciliary activity was recorded at a rate of 500 frames per second (fps) and played back at reduced frame rates for CBP and CBF analysis. CBF was calculated using the following equation [CBF  =  (500/number of frames for 10 ciliary beats) x 10] and a mean CBF was reported from a minimum of 6 measurements on independent ciliated strips. The normal range for mean CBF was 11–20 Hz in Southampton, 10–14 Hz in Leicester. The key differences in method between the centres for CBF measurement are that in Leicester a glass chamber slide is used for the biopsy sample, whereas in Southampton a plastic chamber cassette is used. The microscopy for CBF determination is also slightly different in that Leicester uses a conventional microscope whereas in Southampton an inverted microscope is used. We believe that these differences are unlikely to account for the different normal range data between the two centres. This has been consistent over many years, has been debated at meetings of the UK diagnostics service [19].The UK centres have audited the data and have considered that the differences are genuine but the reasons for the differences have not been identified. This variability did not affect diagnostic outcomes or general agreement on CBF. Static cilia were reported as having a CBF of 0 Hz. In the instance of static and motile cilia being observed together a fast Fourier transform (FFT) algorithm was employed to measure the mean CBF in areas of ciliary beating (in Southampton). CBP was assessed by experienced observers. Dyskinesia was defined as an abnormal ciliary beat pattern that included uncoordinated or asynchronous ciliary beating, reduced beat amplitude, vibratory pattern, hyperfrequent, twitching or static cilia.

**Table 1:** Equipment used at the PCD centres for high speed video microscopy.

	Southampton	Leicester
**Specimen slide**	0.5 mm coverwell imaging chamber (Sigma-Aldrich, Poole, UK) mounted onto a glass slide	Chamber created by the separation of a coverslip and a glass slide by two adjacent coverslips
**Microscopy**	Olympus IX71 inverted microscope with modified condenser. Specimen inverted onto an x100 UPlan wide aperture oil objective.	Leitz Diaplan conventional microscope with x100 interference contrast plan apochromat objective lens.
**Environmental control**	37°C heated environmental chamber (Solent Scientific, Southampton, UK); anti-vibration table.	37°C heated stage of microscope; anti-vibration table (Wentworth Laboratories Ltd, Sandy, UK).
**High speed video imaging and analysis**	Photron FASTCAM MC2 high speed video digital camera and software.	IDT X4 high speed camera. AVI images analysed using MotionPro software, IDT.
**Fast Fourier transform algorithm**	Image J^22^ plugin (P. Lackie, Southampton, UK).	Not used. All readings were done by a trained analyst in a blinded fashion.

### Transmission electron microscopy

Ciliated epithelium for TEM was fixed in 3–4% glutaraldehyde and prepared, imaged and analysed in a blinded fashion as previously described [20]. The percentage of ultrastructural defects was determined by analysis of cilia in cross section. The number of cross sections analysed (typically 300 cilia) was dictated by the size and secondary damage within the fixed sample. Common defects included missing outer and inner dynein arms alone or together, truncated outer dynein arms and microtubular disarrangement.

### Air-liquid interface cell culture

The cell culture methodology was as previously described^16^. In brief, nasal brush biopsies were grown on collagen (0.3 mg/ml, Nutacon, Netherlands) coated tissue culture trays (12 well) in bronchial epithelial growth medium (BEGM) (BEBM including SingleQuots, Lonza, USA) for approximately a week. Confluent basal cells were then expanded into collagen coated flasks (25 or 75 cm^2^) and the BEGM replaced three times per week. Suspensions of the non-ciliated basal cells were then seeded (100,000–500,000 cells per well) onto collagen-coated transwell inserts (1.2 cm^2^) (Costar, Corning, USA) in a 12 well culture plate under ALI medium (1:1 BEBM:DMEM 4.5 g/L D-glucose including SingleQuots) until confluent. The monolayer of primary human basal epithelial cells was then exposed to an air-liquid interface, feeding them basolaterally only with ALI-medium supplemented with an additional 100 nM all-trans retinoic acid (Sigma-Aldrich, UK). The medium was exchanged three times per week and any apical surface liquid or mucus removed. Once cilia were observed (3–6 weeks post transition to ALI), the ciliated epithelium was recovered (by gentle scraping using a spatula) from the transwell for analysis of ciliary function and TEM analysis of the axonemal structure, as described above (Leicester), or a quarter segment of the ALI culture was mounted in the chamber slide, prior to CBF determination (x100 objective lens) by fast fourier transformation analysis (Southampton).

The degree of ciliation per well varied between 5–50% in each subject and the time to ciliogenesis was also variable between 3–8 weeks post ALI culture. There was no difference in the ciliogenesis time between PCD and non-PCD cultures.

### Statistical analysis

CBF was determined from at least 6 separate strips of epithelium sideways on to the observer and the mean and confidence intervals reported for each subject. Data was normally distributed. Paired t-tests were used to compare mean CBF before and after culture, statistical analysis was performed in SPSS version 20 for Windows (IBM Corp., Armonk, NY) and figures produced in GraphPad prism 5 (GraphPad, San Diego, USA). A p-value less than 0.05 was considered significant.

## Results

### Participants

**Figure 1:**
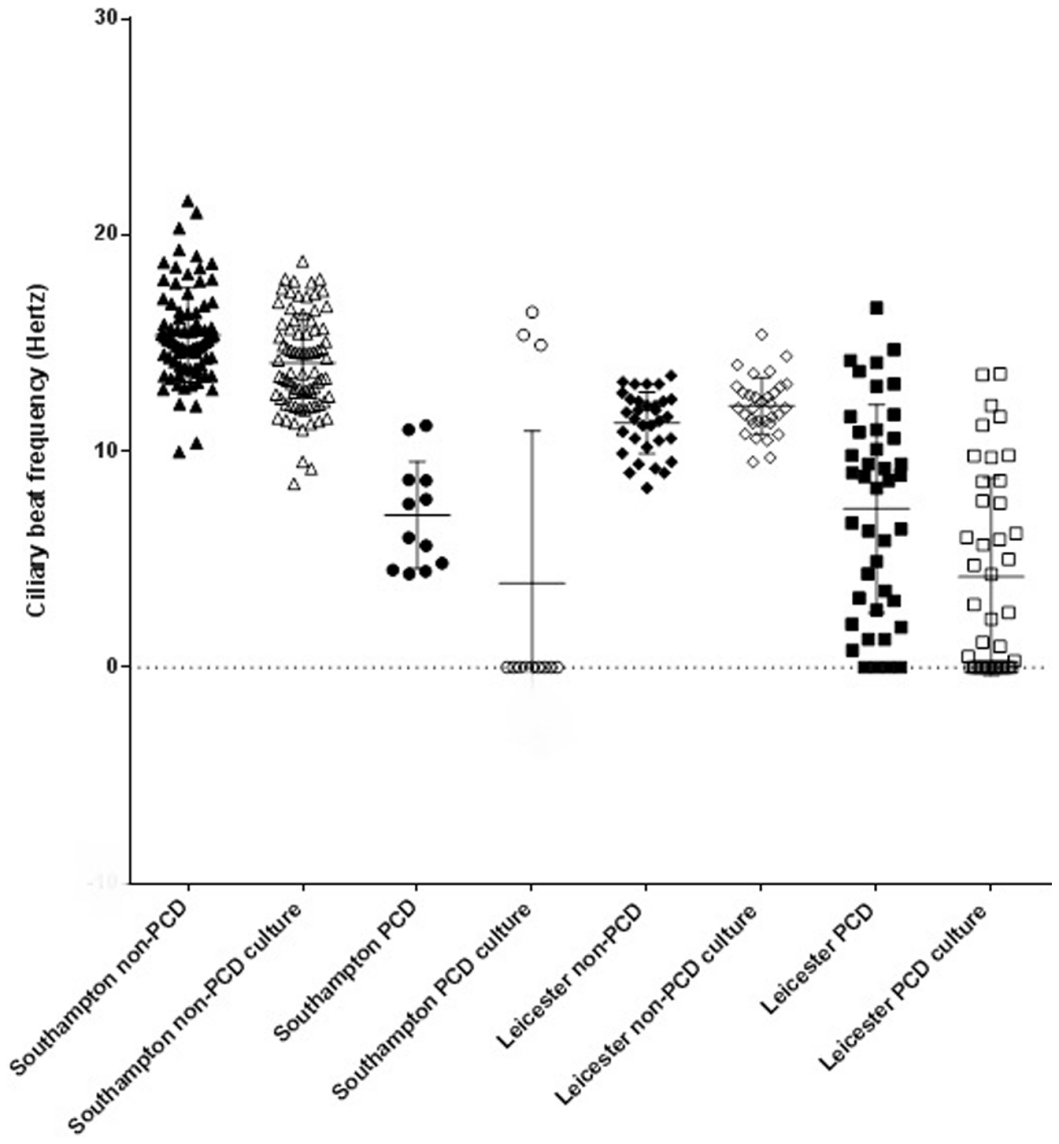
Ciliary beat frequency measured during high speed video analysis of samples before and following ALI culture at the two diagnostic centres. Subjects were diagnosed with PCD or deemed non-PCD based on a portfolio of diagnostic measures.

Participants were patients referred to the national PCD centres in Southampton (n = 92) and Leicester (n = 73) for diagnostic testing. We included consecutive subjects who had paired analysis of ciliary function before and following ALI culture as part of their PCD diagnostic assessment. Seven subjects whose samples were insufficient for analysis but whose post-culture sample was used for diagnostic analysis are not included in the study. Forty-one participants from Leicester and 13 from Southampton were diagnosed with PCD based on a combination of clinical history, ciliary function and ultrastructure^4^ (Table 2). The centres share protocols, however, equipment and subject demographics differ and the two centres have slightly different normal ranges. Following rigorous review across centres we have found that these differences in CBF (but not CBP) did not alter sample processing pathway or ultimately subject diagnosis. Data for the two centres is therefore presented separately as well as combined (Table 3 and Figure 1). All the non-PCD subjects in this study were referred for PCD testing and therefore have upper and/ or lower respiratory symptoms.

**Table 2:** TEM results in subjects with and without PCD.

	PCD		Non-PCD	
	Southampton	Leicester	Southampton	Leicester
**TEM (pre ALI culture)**	OAD + IAD n = 5; OAD n = 2; IAD n = 1; MD n = 1; normal n = 2; equivocal* (60% of inner & outer arms missing) n = 1	OAD+IAD n = 13; OAD n = 4; IAD n = 16; MD n = 4; Transposition n = 4	N = 38. No ultrastructural changes suggestive of PCD.	N = 32 All Normal
**EM (post ALI culture)**	No changes from pre culture except previous equivocal sample, post ALI had 98% of inner & outer arms missing.	No change from pre-culture	N = 9 No ultrastructural changes suggestive of PCD.	N = 32 All normal

Key: TEM =  transmission electron microscopy analysis; OAD =  outer dynein arm defect; IAD =  inner dynein arm defect; MD =  microtubular disorganization defect; ALI = culture at air-liquid interface; PCD =  primary ciliary dyskinesia.

**Table 3:** Ciliary beat frequency (CBF) before and after culture at air-liquid interface in subjects with and without PCD.

	CBF prior to culture (Hz) Range	CBF of ALI cultures (Hz) Range	CBF prior to ALI culture (Hz) Mean (sd)	CBF of ALI cultures (Hz) Mean (sd)	Mean paired difference(95%CI) before and during ALI	*P* value
**PCD Samples**						
Leicester (n = 41)	0 to 16.63	0 to 13.57	7.34 (4.82)	4.20 (4.56)	3.14 (1.84 to 4.44)	<0.001
Southampton(n = 12)$	4.33 to 18.59	0 to 16.44	7.05 (2.48)*	3.89 (7.05)*	3.16 (–0.64 to 6.95)	0.094
Combined	0 to18.59	0 to 16.44	7.28 (4.37)	4.13 (5.15)	3.14 (1.90 to 4.39)	<0.001
**Non- PCD Samples**						
Leicester (n = 32)	8.30 to 13.50	9.5 to 15.40	11.31 (1.42)	12.11 (1.30)	–0.80 (–1.50 to –0.10)	0.027
Southampton (n = 79)	9.97 to 21.6	8.80 to 14.09	15.38 (2.18)#	14.09 (2.24)#	1.29 (0.55 to 2.01)	0.001
Combined	8.30 to 21.60	8.80 to 15.40	14.21 (2.71)	13.51 (2.21)	0.69 (0.10 to 1.27)	0.022

Measurements were made from high-speed video recordings. In samples with mixed static and dyskinetic cilia, a FFT algorithm was used to calculate mean CBF.

$ The data from one Southampton PCD subject was excluded from the analysis because it was an outlier. The cilia of this subject were hyperfrequent and vibrating prior to and following ALI- culture. * = not different compared with Leicester. # = significantly different from Leicester.

### Samples from PCD subjects before and after ALI culture

**Figure 2:**
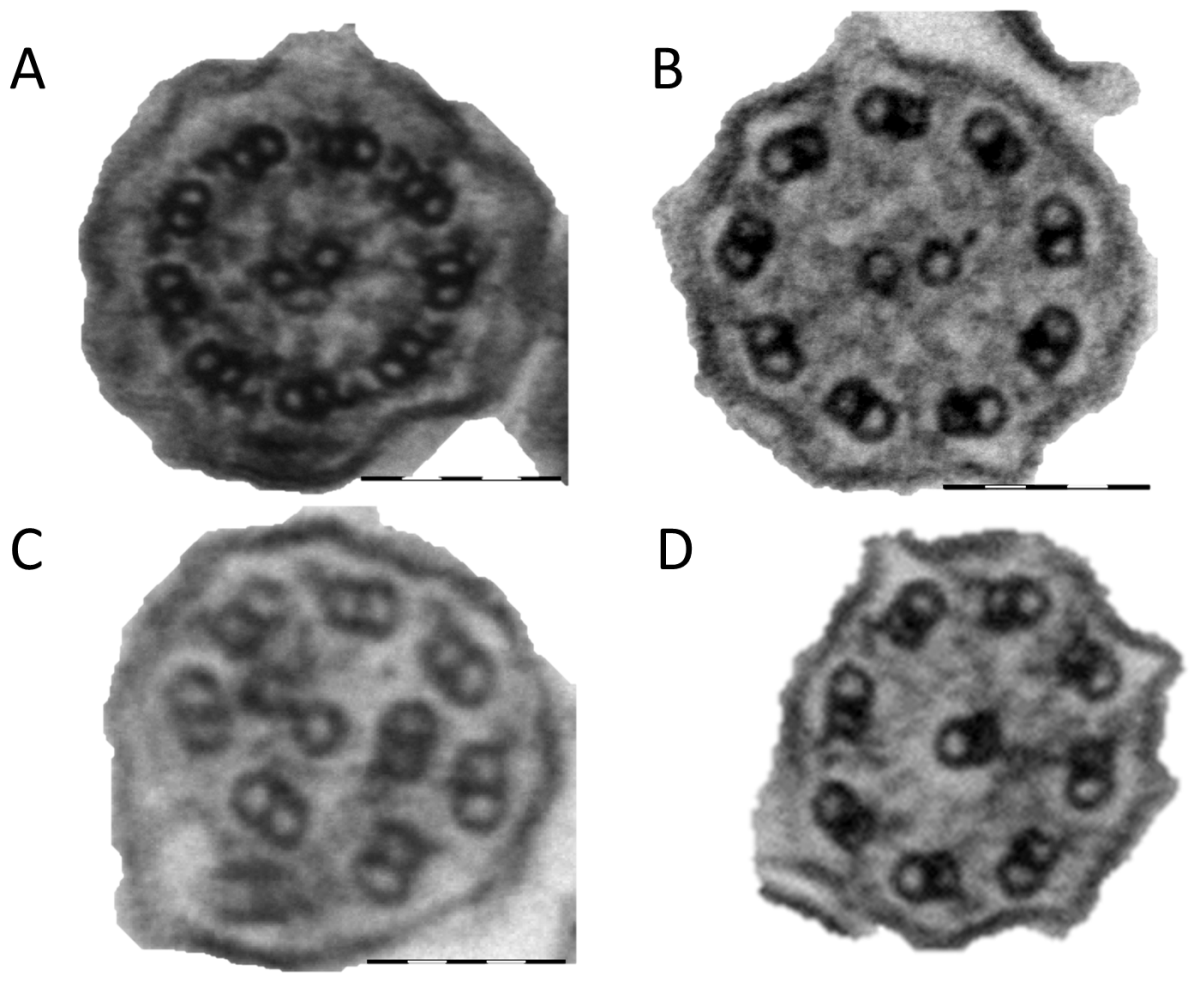
Representative TEM images: a) normal, b) outer and inner dynein arm defect c) microtubule defect d) transposition defect.

The ciliary beat pattern of samples from subjects who were finally diagnosed with PCD was uniformly abnormal prior to culture. Dyskinetic patterns included static cilia, static in combination with twitching cilia, cilia with rigid stroke lacking full sweep and a rotating pattern. Beat frequency was outside the normal range for the majority of PCD cases (Table 3 and Figure 1). All samples were analysed by TEM analysis using samples taken prior to culture. Based on our standard diagnostic criteria, 96% of these had ultrastructural defects associated with PCD (Table 2; Figure 2); 18 had both inner and outer dynein arm defects, 6 had outer dynein arm defects, 17 inner arm defects, 5 had microtubular disorganization, 4 had transposition defects and 2 had normal ciliary ultrastructure. One subject had an equivocal result prior to culture.

Eight of 13 Southampton PCD samples and 13 of 41 Leicester PCD samples demonstrated a combination of static and abnormal motile cilia pre-culture, however post- culture at ALI went on to exhibit completely static or near static (<1 Hz) cilia (supplementary data: video 1 a and b). An additional 9 samples with slow ciliary beat frequency had further reduction of the frequency following culture. All samples continued to demonstrate universal dyskinesia after culture. The mean* ciliary beat frequency decreased following culture (pre-ALI; mean 7.28 Hz; sd 4.37, post-ALI: mean 4.13 Hz sd 5.15; p<0.001) (Table 3). (* Data from one PCD subject was excluded from grouped analysis because it was phenotypically a different variant of PCD. The cilia of this subject were hyperfrequent and cilia appeared to vibrate with reduced range movement prior to and following ALI-culture).

TEM results remained unchanged in all but one case where analysis was repeated post ALI culture (Table 2). One sample from Southampton did demonstrate a change in ciliary ultrastructure following culture. This subject’s pre-culture functional analysis demonstrated a mean CBF of 8.3 Hz ± 0.6 with mixed static cilia and cilia with abnormal motility (100% dyskinetic), which became nearly static (<1 Hz) cilia following ALI culture. TEM analysis of this subject’s cilia prior to culture demonstrated 60% dynein arm defects (loss of both inner and outer dynein arms) which increased to 98% following ALI culture.

The ultrastructural defects that were present in the subjects whose ciliary function changed following culture were both inner and outer dynein arm defects (n = 9), outer dynein arm defects (n = 5), inner dynein arm defect (n = 12) and microtubular disorganization (n = 3). The outer and inner and the outer dynein arm defects had the least ciliary movement compared with the inner arm and microtubular disorganisation group before culture. The degree of motion seen in these EM phenotypes was decreased following ALI culture and there was no individual EM defect that was more prone to becoming static after ALI culture.

### Samples from subjects without PCD, before and after ALI culture

Samples from subjects who finally had the diagnosis of PCD excluded, demonstrated a range of functional ciliary abnormalities when analysed immediately after nasal brushing. These included areas of poor coordination, lack of full sweep and mucus impedance (example of poor coordination: supplementary data file: video 1). The mean ciliary beat frequency of most, but not all samples was within the normal range before culture (Figure 1). TEM analysis demonstrated a variable number of secondary changes (e.g. disrupted membranes, compound cilia) but none had axonemal abnormalities suggestive of PCD.

Following culture, all subjects were considered not to have PCD on the basis of normal post-ALI culture ciliary function (supplementary data file: video 2), combined with other diagnostic criteria including TEM. The mean ciliary beat frequency tended to decrease following culture in the Southampton cohort only, (pre-ALI mean 14.21 Hz, sd 2.71; post-ALI mean 13.51 Hz sd 2.21; p = 0.02) but the size of this drop in frequency was small and not considered physiologically relevant (mean paired difference 0.69 Hz; 95%CI 0.10 to 1.27) (Table 3). Samples from individuals who did not have PCD did show some changes in CBF but these were changes into the normal range. On occasions, some individuals displayed an increased or decreased CBF outside of the normal range that became normal following ALI-culture.

Ultrastructure was re-analysed by TEM post-culture in 41 non-PCD samples. These continued to show no ultrastructural defects suggestive of PCD.

### Cilia Beat Pattern (CBP) and nasal Nitric Oxide

CBP and CBF was determined in all samples including the fresh biopsy and post cultured cells. In non-PCD subjects the dyskinesia was significantly reduced after cell culture (supplementary Video S1 and S2) In Leicester 65±25% were dyskinetic pre-culture and 55±25% post culture. In Southampton, 85% of strips analysed from non-PCD patient included dyskinetic cilia pre-culture but following ALI-culture in each case over 75% of ciliated cells demonstrated normality. The PCD subjects from both centres displayed 100% abnormal cilia that showed a dyskinetic or static beat pattern before and after culture (supplementary Video S3 and S4). Nasal nitric oxide determination was performed at both centres but only in a proportion of the subjects (due to age or ability to do the manovre).The non-PCD subjects had a mean nNO of 162±78ppb (Leicester), 570±350ppb whereas the PCD subjects had a significantly lower nNO of 23±19ppb (Leicester), 34±19ppb (Southampton).

## Discussion

This is the largest study to evaluate analysis of ciliary function and ultrastructure before and after air-liquid interface culture. It confirms that post-culture changes in the phenotype of PCD samples clarifies rather than confuse diagnostic status. Prior to the study, Southampton and Leicester PCD Centres simultaneously and independently recognised exacerbation of the functional defects seen in some subjects with PCD following ALI culture. This led to concerns about the validity of the technique. However, this large study cohort of 54 PCD subjects and 111 non-PCD subjects reassures us that the post-culture changes actually aid diagnosis. In all cases dyskinesia associated with PCD was unchanged or became more prominent. Secondary dyskinesia in the non-PCD group improved. Cell culture, is a highly specialised technique and our success rate of culturing nasal ciliated epithelium from subjects using at an air liquid interface method ranges from 54% from all biopsies up to 79% for biopsies with a low level of dyskinetic cilia. This is a lower success rate than is attainable using a simple submerged culture technique, but has the advantage of yielding more, longer lived cilia, sufficient for post culture ultrastructural analysis.

One of our findings was that in some subjects with PCD, the CBF was within the normal range. This demonstrates that the CBF alone cannot be a reliable determinant of PCD. The CBF reading is normal in certain phenotypes of PCD and assessment of CBP is required alongside the CBF for PCD diagnosis.

The reason for the PCD cilia movement becoming slower in some subjects is unknown but we can speculate. Post-culture functional changes were seen in subjects with a variety of ultrastructural defects, including outer dynein arm defects, combined inner and outer arm defects, inner arm defects, transpositions, and microtubular disorganization defects. This makes a genetic association less likely, although one could speculate that an environmental condition was having a more general effect on gene expression.

We considered whether the culture was preferentially selecting or cloning cells that demonstrated static cilia, but on review of the images, static and twitching cilia were generally co-located on cells in the original brush biopsies. We wonder whether the absence of any smooth muscle and nervous innervation in the artificial ALI cultures may pay a role in this phenomenon as pharmacologically active agents have been shown to regulate CBF [21]. We could also speculate that secretions in the ALI are different and that endogenous release of ciliary stimulation factors may be reduced in PCD ALI cultures. In culture, the local reinforcement of beat frequency and pattern due to mass flow of material along the airways may be reduced compared to *in vivo*, increasing the expression of dyskinetic patterns and possibly permitting the survival of cells that may be lost *in vivo.*

This study has confirmed that reanalysis following ALI-culture is useful where the original brush biopsy health was poor. Damage due to infection, inflammation or trauma during sampling causes secondary dyskinesia commonly making diagnostic status uncertain. Reanalysis of newly differentiated cilia in epithelial cell cultures in the non-PCD cohort confirmed normal ciliary frequency and beat pattern, enabling us to confidently exclude the diagnosis of PCD. ALI-culture has the additional benefit of providing sufficient ciliated epithelium for TEM analysis of the ciliary ultrastructure. TEM is an essential part of the diagnostic testing for PCD, but our protocols require analysis of 300 healthy cilia in cross section. From Southampton, 7 subjects had insufficient cilia for a full analysis on the original brushing, but we were able to complete the analysis using the ALI sample. Without using ALI-culture, we would have needed to recall a sizable percentage of subjects for repeat nasal brushing biopsy for further ciliary function analysis, TEM or both.

In addition to the diagnostic benefits of this technique, ALI-culture and submerged-culture of primary epithelial cells has proved excellent models facilitating research into PCD [22] and other [23], [24] respiratory diseases. A limitation of this study is that it is retrospective. The fact that it was conducted at two centres which shared protocols but had different equipment has its strengths and limitations.

In summary, respiratory epithelial cells obtained by nasal brush biopsy showed phenotypic changes following culture at air-liquid interface, including reduced CBF, which facilitated diagnosis or exclusion of PCD. The phenotype of PCD appeared enhanced in a number of subject samples following re-growth using ALI-culture.

## Supporting Information

Video S1 **Ciliary activity before and after ALI culture from non-PCD subject showing secondary dyskinesia.** Recorded at 500fps and played at 30fps.
(MPG)

Video S2 **The same subjects cilia as in Video S1 with improvement of secondary dyskinesia following culture.** The image was recorded at 500fps and played back at 30fps.
(MPG)

Video S3 **High speed video images of ciliary function from a subject with PCD showing abnormal movement.** Recorded at 500fps and played at 30fps.
(MPG)

Video S4 **The same PCD subject as in Video S3 showing all of the cilia are static following culture.** Recorded at 500fps and played at 30fps.
(MPG)
